# Disseminated fusariosis in a patient with bone marrow aplasia^☆^^☆☆^

**DOI:** 10.1016/j.abd.2019.12.008

**Published:** 2020-07-04

**Authors:** Danielle Ferreira Chagas, Lucia Martins Diniz, Elton Almeida Lucas, Paulo Sergio Emerich Nogueira

**Affiliations:** Dermatology Service, Hospital Universitário Cassiano Antonio Moraes, Universidade Federal do Espírito Santo, Vitória, ES, Brazil

**Keywords:** Fusariosis, Immunosuppression, Mycoses, Neutropenia

## Abstract

Fusariosis is a superficial or systemic infection, which occurs mainly in immunocompromised hosts, especially in patients with hematological neoplasia; 70%–75% of the cases present cutaneous manifestations. The disseminated form is rare and difficult to diagnose; even with specific treatment, the evolution is usually fatal. Currently, it is considered an emerging disease; in some centers, it is the second most common cause of invasive mycosis, after aspergillosis. The authors describe a case of a female patient with idiopathic bone marrow aplasia and disseminated fusariosis, who initially appeared to benefit from voriconazole and amphotericin B; however, due to persistent neutropenia, her clinical condition deteriorated with fatal evolution.

## Introduction

Fusariosis is an opportunistic, cosmopolitan disease caused by filamentous, hyaline fungi of the *Fusarium* genus, widely distributed in nature as soil and plant saprobes.^1^ It rarely affects immunocompetent individuals; when it does, the infection usually remains superficial, causing onychomycosis and keratitis, related to direct inoculation.^2^, ^3^

In immunocompromised patients, especially those with hematological cancer, in particular acute myeloid leukemia, and after bone marrow transplantation, invasive fungal infections are associated with 70% mortality.^4^ In disseminated infections, 80% of patients develop skin lesions, which may be the only early manifestation of the disease.^2^, ^5^

In its disseminated form, fusariosis is a rare infection, with an incidence of 0.06% to 0.2% in the United States and Europe; in hematological patients, however, it is associated with high morbimortality, due to the increased incidence and the low effectiveness of treatments.^3^, ^6^

This report describes a case of disseminated fusariosis with cutaneous involvement in an immunocompromised patient due to bone marrow aplasia.

## Case report

Female patient, 29 years old, previously healthy, admitted to a tertiary hospital due to the sudden onset of petechiae in the lower limbs two months before, associated with fever of recent onset. On admission, she was diagnosed with pancytopenia and severe febrile neutropenia (neutrophils below 100 cells/mm^3^), and broad-spectrum antibiotic therapy (meropenem and vancomycin) was initiated.

In the investigation, a bone marrow biopsy was performed; the histopathology showed bone marrow hypoplasia of the three hematopoietic cell lines, with only 5% of cells. In addition, all serologies (including parvovirus B19) were requested, and all infectious hypotheses were discarded; therefore, the diagnosis of idiopathic bone marrow aplasia was reached.

The patient had persistent fever despite antibiotic therapy, but without bacterial growth in blood cultures. Due to hemodynamic instability, the patient was taken to the intensive care unit, and amphotericin B was indicated at a dose of 5 mg/kg/day, due to febrile neutropenia unresponsive to antibiotic therapy.

**Figure 1:**
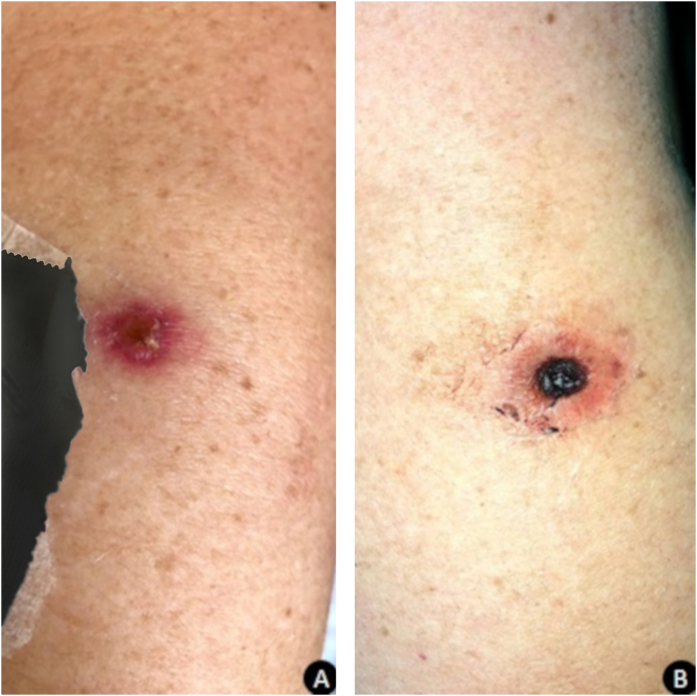
A, An erythematous-violaceous macula on the left upper limb. B, After one week, the lesion evolved to ulceration with a necrotic center and slight erythema around it.

**Figure 2:**
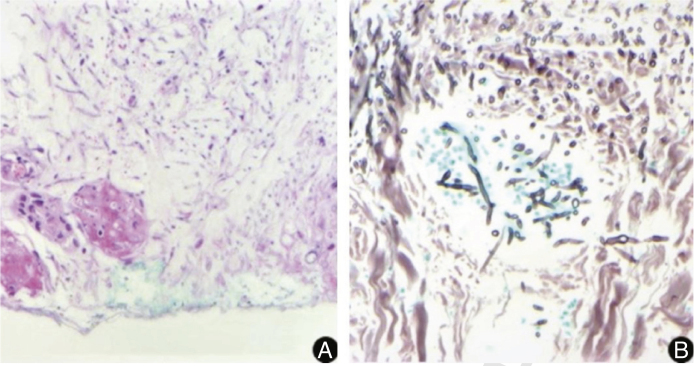
Histopathological examination of a lesion of the left upper limb, showing multiple septate, hyaline, and branched hyphae with angiolymphatic invasion. (A, Hematoxylin & eosin, ×10; B, Grocott, ×40).

**Figure 3:**
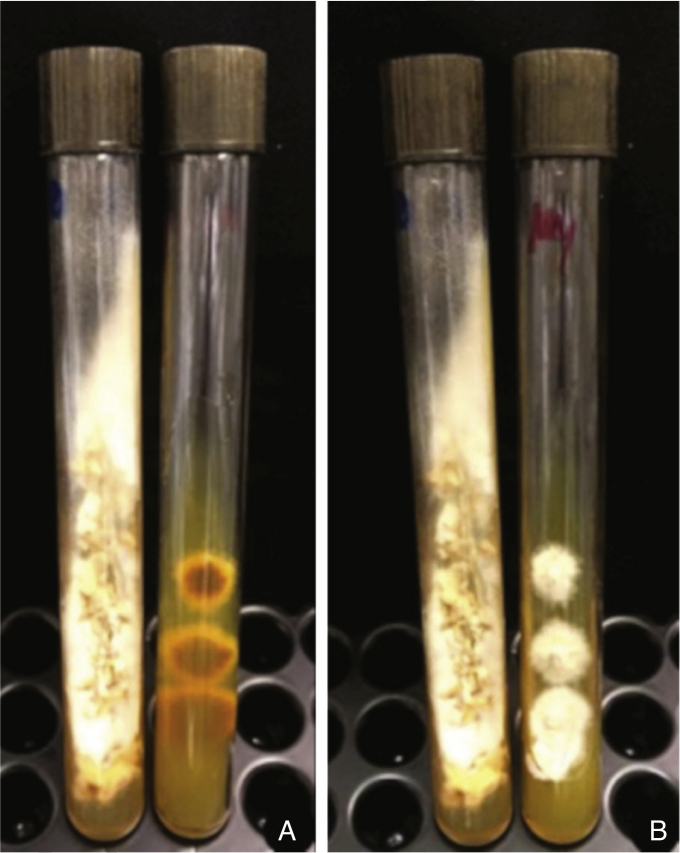
A, Skin fragment culture in Sabouraud’s medium with chloramphenicol: white powdery filamentous colony; B, Lilac-colored pigmentation on the reverse side.

**Figure 4:**
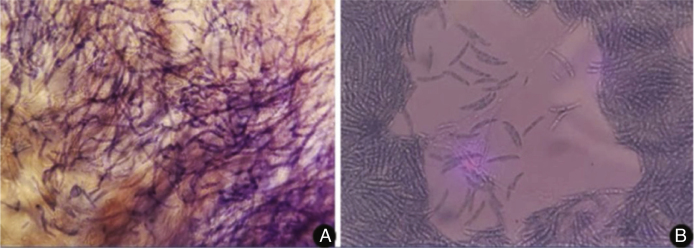
A, Direct examination of the skin lesion showing a large number of filamentous fungi; B, Microculture of the colony stained by lactophenol cotton blue, with macroconidia featuring characteristic canoe-type morphology.

After the seventh day of hospitalization, the patient presented a painful, erythematous-violet macula in the left upper limb, which after one week evolved with central necrosis (Fig. 1). A lesion biopsy was performed; the histopathology was compatible with leukocytoclastic vasculitis and numerous hyphae were seen on the vessel wall (Fig. 2). Direct examination of material from the skin lesion revealed filamentous fungi. Culture in Sabouraud’s medium with chloramphenicol from the skin fragment and subsequent microculture of the colony evidenced the growth of *Fusarium* spp. (Figure 3, Figure 4). After the results of these tests, amphotericin B was associated with voriconazole, and the patient initially benefited from this association.

**Figure 5:**
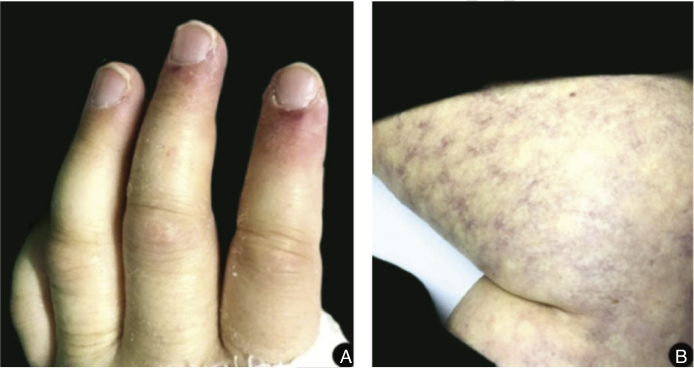
A, Paronychia in the second and third left fingers; B, Exuberant livedo reticularis affecting the entire lower limb.

The dermatological examination also showed paronychia in the second and third left fingers and exuberant livedo reticularis in all lower limbs, extending to the abdomen (Fig. 5).

**Figure 6:**
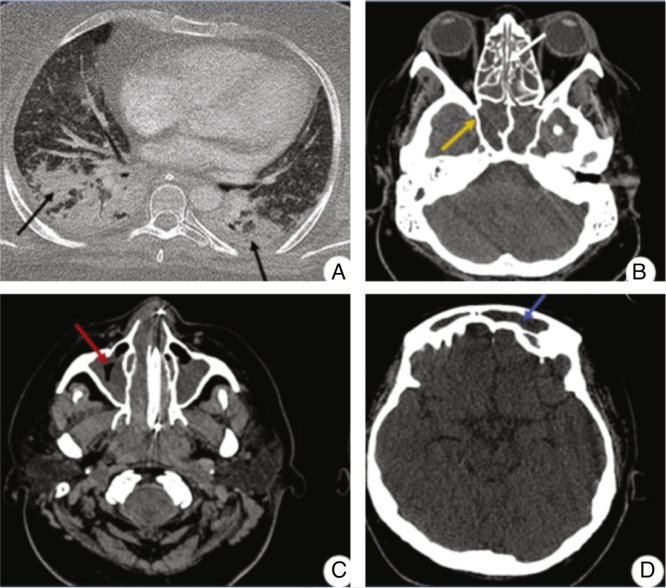
A, Chest tomography showing a pattern of parenchymal consolidation with intermingled air bronchograms, notably in the posterior region of the pulmonary fields (black arrows). (B, C, and D) CT scan of the sinuses showing diffuse veiling of the paranasal sinuses, characterized by material with soft tissue density filling the maxillary (red arrow), sphenoid (yellow arrow), and frontal sinuses (blue arrow), and the ethmoidal cells (white arrow).

The image examination of the sinuses revealed extensive shadowing of the maxillary, frontal, sphenoid, and ethmoid sinuses, which was attributed to invasive sinusitis (Fig. 6). The otorhinolaryngology team performed a biopsy of the sinuses and a direct examination of the nasal cavity. Surgical treatment through debridement was not possible due to persistent thrombocytopenia and lesion angioinvasion. Histopathology and direct examination were similar to the findings of the skin lesion. She also had extensive bilateral diffuse pulmonary infiltrate consistent with invasive pneumonia.

Throughout the period of neutropenia, the patient received transfusions and stimulating factor for granulocytic colonies, but did not present any bone morrow response. After four weeks, she developed refractory septic shock and, despite supportive measures, died 54 days after hospitalization.

## Discussion

Fusariosis is the second most frequent invasive fungal infection in patients with hematological neoplasms; *Fusarium solani* is the most common, virulent, and resistant species, presenting the highest mortality, confirmed in the case presented.^6^, ^7^, ^8^

The infection starts by inhaling conidia or by direct contact with materials contaminated by spores.^1^, ^2^ Studies indicate that disseminated cases are usually acquired by inhalation with subsequent dissemination to other organs such as the kidneys, liver, eyes, spleen, and brain.^1^, ^8^ In the reported case, skin lesions preceded sinusitis and pneumonia, suggesting hematogenous spread of cutaneous focus. The infection is classified as disseminated when two or more organs are involved, as seen in the case reported, in which the patient presented sinusitis and pneumonia, confirmed by image examination and the presence of skin lesions.^4^

The most common presentation is persistent fever unresponsive to broad-spectrum antibiotic therapy in a neutropenic patient, such as the patient studied. Typical skin involvement shows painful erythematous-violet macules or papules, the center of which evolves to necrosis, usually on the extremities; all these findings were observed in the present case. The dermatological examination also showed livedo reticularis in the entire lower limb reaching the abdomen, which probably occurred due to intravascular proliferation of the fungus leading to occlusion and necrosis of the microvasculature, although the authors have not found any reports of this phenomenon in the literature.^5^, ^8^, ^9^

The diagnosis requires the isolation of *Fusarium* spp.^5^ In the present case, hyphae were observed in the histopathology of the skin and sinuses, confirmed by the growth of fungi in the culture of samples collected at these sites. In histopathological examination, fungi characteristically present angiolymphatic invasion by septate, hyaline, and branched hyphae.^1^ Culture identification is important to help differentiate fusariosis from other hyalohyphomycoses. The *Fusarium* genus is identified in the culture by multiple canoe-shaped hyaline macroconidia.^6^, ^7^ However, species identification requires molecular methods.^7^

Invasive and generalized infections respond poorly to antifungal therapy, partly due to drug resistance, but mainly due to the lack of an effective response from the host, which led to our patient's unfavorable outcome, who remained with persistent neutropenia. Therefore, treatment is based on systemic antifungals and reversal of immunosuppression. The ideal treatment should be guided by the antifungal sensitivity test, which is available in only a few centers; therefore, most authors recommend combined therapy for severe cases, with voriconazole and amphotericin B, the scheme used in the present patient.^2^, ^8^, ^10^

The patient had severe neutropenia related to bone marrow aplasia, and the authors found in the literature three cases of disseminated fusariosis associated with this hematological disease, all of which also had a fatal outcome.

## Financial support

None declared.

## Authors’ contributions

Danielle Ferreira Chagas: Conception and planning of the study; elaboration and writing of the manuscript; intellectual participation in propaedeutic and/or therapeutic conduct of studied cases; critical review of the literature.

Lucia Martins Diniz: Approval of the final version of the manuscript; conception and planning of the study; effective participation in research orientation; intellectual participation in propaedeutic and/or therapeutic conduct of studied cases; critical review of the literature; critical review of the manuscript.

Elton Almeida Lucas: Intellectual participation in propaedeutic and/or therapeutic conduct of studied cases.

Paulo Sergio Emerich Nogueira: Intellectual participation in propaedeutic and/or therapeutic conduct of studied cases.

## Conflicts of interest

None declared.
